# What is the most appropriate method for coronary sinus cannulation? The telescopic method or the electrophysiologic method?

**DOI:** 10.1371/journal.pone.0203534

**Published:** 2018-09-14

**Authors:** Hakan Gunes, Ekrem Aksu, Huseyin Nacar, Murat Kerkutluoglu, Handan Gunes, Sami Ozgul

**Affiliations:** 1 Department of Cardiology, Sutcu Imam University, Kahramanmaras, Turkey; 2 Department of Cardiology, Adıyaman University, Adıyaman, Turkey; 3 Department of Physiology, Cumhuriyet University, Sivas, Turkey; Faculty of Medical Science - State University of Campinas, BRAZIL

## Abstract

**Objectives:**

The most challenging stage of cardiac resynchronization therapy (CRT) is coronary sinus cannulation (CS). The aim of this study was to compare coronary sinus cannulation techniques using electrophysiology catheters and coronary angiography catheters.

**Methods:**

In this observational, retrospective and non-randomized study, 87 patients who were eligible for CRT device implantation were screened at Kahramanmaras Sutcu Imam University Hospital between March 2014 and March 2018. Seventy-two patients who met the inclusion criteria were enrolled in the study. The study population was divided into 2 groups: the first group consisted of 36 patients whose coronary sinuses were cannulated via electrophysiology (EP) catheters and the second group included 36 patients who received coronary angiography catheters for coronary sinus cannulation.

**Results:**

The two groups were similar in terms of the baseline characteristics of the patients. The total fluoroscopy time was less with cannulation using coronary angiography catheters. There were no differences between the two groups in terms of the amount of contrast material and the success of the operations.

**Conclusions:**

Coronary sinus catheterization using coronary angiography catheters significantly reduces fluoroscopy time in patients undergoing CRT.

## Introduction

Cardiac resynchronization therapy (CRT) is recommended for patients with New York Heart Association (NYHA) Class II-IV symptoms, sinus rhythm, ejection fraction below 35%, and QRS duration ≥150 ms with morphology of left bundle branch block (LBBB) or non-LBBB, despite optimal medical therapy [1–10]. It has been shown to improve cardiac performance in selected patients and decrease morbidity and mortality [11–12].

The procedure used for cardiac resynchronization therapy is similar to the procedure used for the implantation of conventional pacemakers and defibrillators. Differently, a left ventricular electrode is required for cardiac resynchronization therapy. It is usually placed via the coronary venous approach because the left ventricular electrode is less invasive. Despite the difficulties caused by the very variable anatomic structure of the coronary venous system, the success rate of left ventricular electrode implantation by means of cannulation of the coronary venous system is over 95% for experienced operators. The high rate of successful implantationis based on the following recommendations: good knowledge of the anatomy of the coronary venous system; having good visualization of the coronary venous system prior to and during the operation; and selecting the appropriate catheter and method for the appropriate coronary venous system cannulation based on the obtained images [13–15].

The aim of this study was to compare two different methods for coronary sinus cannulation, an important step in cardiac resynchronization therapy.

## Method

In this observational, retrospective, and non-randomized study, 87 patients aged over 18 years who underwent CRT device implantation for heart failure with reduced ejection fraction with ischemic or non-ischemic etiology were screened between March 2014 and March 2018 at Kahramanmaras Sutcu Imam University Hospital. It was confirmed that the indications for CRT were in accordance with current European Society of Cardiology guidelines for all patients [16].Four of the 87 patients were excluded because coronary sinus images were obtained by coronary angiography before cannulation and 2 patients were excluded due to chronic renal failure. In addition, the time to coronary sinus cannulation was not available in 5 patients. Accordingly, 11 of the 87 patients were not included in the study. The remaining 76 subjects were divided into 2 groups: the first group consisted of 36 patients whose coronary sinuses were cannulated using electrophysiology catheters, and the second group included 40 patients who received coronary angiography catheters for coronary sinus cannulation. Four patients in the second group underwent an upgrade from implantable cardioverter defibrillator (ICD) to cardiac resynchronization therapy defibrillator (CRT-D) systems; therefore, they were also excluded from the study. Thus, a total of 72 subjects with 36 patients in each group were examined in this study. All implantation procedures were performed by the electrophysiology team of our clinic, which comprises 4 electrophysiologists and performs more than 20 successful coronary sinus cannulations per year. In both groups, right ventricle, left ventricle, and right atrium leads were placed, respectively.

A routine pacemaker lead-placement procedure was made for right ventricular and right atrial electrodes. In the first group (EP catheter-mediated method—group 1), device implantation was performed by electrophysiologists using an ablation catheter for coronary sinus cannulation. After the subclavian venous puncture, the coronary sinus cannulation catheter (CPS Direct™ PL Peelable Outer Guide Catheter ST-JUDE) was guided through the guidewire to the coronary sinus mouth (CPS direct™ PL Peelable Outer Guide Catheter ST-Jude), then the coronary sinus was cannulated in the left anterior oblique position by passing the RFMarinr (Mc Multi-Curve Steerable Ablation Catheter) through the cannulation catheter. If cannulation was not successful with the RF Marinr, cannulation was performed using a Livewire TC (bi-directional ablation catheter). If cannulation could not achieved with both catheters, it was evaluated as a failure of the method, then another technique (telescopic method or classic method) or coronary angiography-guided coronary sinus imaging was used. The device implantation of patients in the second group (telescopic method-group 2) was performed by electrophysiologists using a coronary angiography catheter for coronary sinus cannulation. After the subclavian venous puncture, the coronary sinus cannulation catheter (CPS Direct™ PL Peelable Outer Guide Catheter, St. Jude) was extended through the guidewire to the coronary sinus ostium, angiographic catheters with different angles were passed through the AL2, MultiPurpose or right-guiding cannulation catheter (Launcher coronary guide catheter) to perform different maneuvers in the left anterior oblique position (generally clockwise reversal), then small contrast injections were delivered to the coronary sinus. The first choice was AL2, but if success was not achieved with this catheter, cannulation was performed using a right-guiding and MP catheter according to the shape of the coronary sinus ostium. In the event of failure to perform the cannulation with these catheters, the case was evaluated as a failure of method, then another technique (EP catheter or classic method) or coronary angiography-guided coronary sinus evaluation was used. After all methods had been tried, failure to perform cannulation was defined as failure of the procedure. The amount of contrast material used, total fluoroscopy time, the percentage of procedure success, and the methods used in both groups were recorded for all patients. Following CRT implantation, the presence of any complications such as pneumothorax, hemothorax, and pocket hematoma was recorded. After 10 days, the incision area was reevaluated during the removal of the sutures, and any complication such as wound infection was recorded.

The study was performed in accordance with the Declaration of Helsinki for Human Research, and was approved by Kahramanmaras Sutcu Imam University Local Ethics Committee (Protocol code:269; Decision no:19: Date: 03/01/2018).

### Statistical analysis

Statistical analysis was performed using the SPSS version 14.0 software (SPSS Inc., Chicago, IL, USA). The Kolmogorov-Smirnov test was used to confirm the normality of the distribution of continuous variables. Continuous variables are expressed as mean ± SD or median (min-max) in the presence of non-normal distribution, and categorical variables as percentages. Comparisons between the groups were made using the Chi-square or Fisher’s exact tests for categorical variables, independent samples t-test for normally distributed continuous variables, and the Mann-Whitney U test when the distribution was skewed. A p-value of 0.05 was considered as statistically significant.

## Results

The mean age and body mass index (BMI) of the group undergoing coronary sinus cannulation with the EP catheter-mediated method (group1) were 65±8 years and 29±5 kg/m^2^, respectively; 25% of these patients had ischemic cardiomyopathy. The mean age and BMI of the group undergoing coronary sinus cannulation with the telescopic method (group2) were 63±9 years and 27±5 kg/m^2^, respectively; 30% of these patients had ischemic cardiomyopathy. The basal characteristics, echocardiographic and laboratory characteristics were similar in the two groups (Table 1). Successful cannulation was achieved with EP catheters in 33 of the 36 patients. Thirty-two of these patients were cannulated with RF-Marinr, and due to failure to provide cannulation with RF Marinrfor one of the patients, cannulation was performed using Livewire TC as a second alternative. The telescopic method was tried in 3 patients because of failure of cannulation with the EP catheter (percentage of crossing-over: 8%).One of these patients was cannulated with AR2 coronary catheters. The other two patients were not cannulated despite attempting all the methods, so a double-chamber ICD was implanted. Thirty-four of the 36 patients who underwent coronary sinus cannulation with the telescopic method were successfully cannulated. Thirty-one of the 34 patients were cannulated using only AR2 coronary catheters. In 3 patients, right-guiding catheters were used for cannulation due to failure of catheterization with AR2s. Two patients in this group were not able to be cannulated with coronary catheters (AR 2 right-guiding and MPs were tested). These two patients were cannulated using RF Marinr, thus there were no non-cannulated patients (percentage of crossing-over: 6%).Tricuspid insufficiency and dilatation presence in right heart chambers were similar in both groups.

**Table 1:** Baseline characteristics of study patients.

	EP catheter-mediated method (group-1) (n:36)	Telescopic method (group-2) (n:36)	p
**Baseline characteristics**			
Age (years)	65±8	63±9	0.309
Height (cm)	157±6	160±9	0.044
Weight(kg)	72±13	72±10	0.937
BMI(kg/m2)	29±5	7±5	0.355
Male/female	26/10	21/15	0.322
Hypertension (%)	26 (72%)	19 (58%)	0.144
Diabetes mellitus (%)	19 (53%)	14 (%)	0.344
Current smoking (%)	15 (42%)	14 (39%)	1.000
Hyperlipidemia (%)	27(75%)	19(53%)	0.086
COPD (%)	9 (25%)	11(31%)	0.792
Coronary artery disease (%)	9 (25%)	11(31%)	0.792
**Echocardiographic findings**			
Left atrial diameter (cm)	3.8 ±0.6	3.6 ± 0.5	0.183
LV ejection fraction (%)	31±4	32 ±4	0.173
LV diastolic diameter (cm)	5.1±0.9	4.9±0.8	0.351
Presence of RV dilatation	3 (9%)	4 (11%)	1.000
Presence of severe TR	3 (9%)	4 (11%)	1.000
**Labaratory findings**			
Hemoglobin (gr/dl)	12.9±2.2	12.6±2.0	0.567
Platelets counts(10^3^)	243±92	221±70	0.255
BUN (mg/dl)	16 (10–25)	20 (10–95)	0.082
Creatinine (mg/dl)	1.0 (0.5–5.7)	1.0 (0.5–6.5)	0.345
Potasium (mmol/l)	4.5±0.5	4.5±0.4	0.875
Sodium (mmol/l)	137±3	136±3	0,084
Fasting glucose (mg/dL)	148±60	138±67	0.493

BMI: Body Mass İndex, COPD:Chronic obstructive pulmonary disease,LV: Left ventricle, RV: Right ventricle, TR: Tricuspit regurgitation

Data are presented as mean ± SD, number and percentage, or median (min-max) and range. P ≤0.05 was considered statistically significant.

According to the success rates of the procedures, a 92% success rate was achieved with EP catheters and 94% with the telescopic method (p = 0.999). The amount of contrast agent used was similar in the two groups [30 (range, 20–45) ml vs. 33 (range, 15–55) ml, p = 0.159]. When assessed from the point of view of fluoroscopy, it was found that the duration of fluoroscopy in the telescopic method was significantly shorter when compared with the EP catheter-mediated method (28±13 minutes vs. 20±10 minutes, p = 0.004). Procedure-related complication rates were similar between the two groups (Table 2). No wound infections were observed in any patients.

**Table 2:** Primary and secondary endpoints in EP catheter-mediated method vs. telescopic method.

	EP catheter-mediated method (group-1) (n:36)	Telescopic method (group-2) (n:36)	p
Total fluoroscopy time (min)	28±13	20±10	0.004
Contrast agent amount (ml)	30 (20–45)	33(15–55)	0.159
Success (%)	33 (92%)	34(94%)	1.000
Incidence of pocket hematoma (%)	3(8%)	2 (6%)	1.000
Incidence of pneumothorax (%)	1(3%)	0	1.000
Incidence of hemothorax (%)	0	1(3%)	1.000
Percentage of crossing-over (%)	3(8%)	2(6%)	1.000

Data are presented as mean ± SD, number and percentage, or median (min-max) and range. P ≤0.05 was considered statistically significant.

## Discussion

Coronary sinus cannulation is an important stage of CRT device implantation. It is of great importance in both coronary sinus imaging and the placement of the left ventricular electrode in the appropriate region. In addition, coronary sinus cannulation is the greatest cause of procedure failure; it is also the most time-consuming and most challenging aspect [13–17]. For this reason, different techniques have been used for coronary sinus cannulation. In some compelling cases, new techniques have been used to provide coronary sinus cannulation, but in general, 3 techniques are used: standard cannulation with coronary sinus placement catheters, EP catheters, and with coronary catheters [18, 19]. Although the frequency and success of these techniques vary according to the operator, standard cannulation with a coronary sinus catheter is usually performed. As with all invasive procedures, one of the most important factors to consider in CRT implantation is radiation exposure. Shortening the fluoroscopy time is as important as the success of the procedure. Radiation exposure has been reduced with the use of sensor-based navigation systems [20]. However, the difficulty of implementation and accessibility of these systems are important problems. Er et al. compared the use of standard catheters and EP catheters in their study and showed that the fluoroscopy duration of cannulation using EP catheters was shorter. They also showed that there was no difference between the two groups in terms of procedure success [21]. In our study, the EP catheter-mediated method and the telescopic method used for coronary sinus cannulation were also compared, and the fluoroscopy time was shorter than in cannulations using coronary catheters. In our study, there was an eight-minute difference between the two groups with regard to the duration of coronary sinus cannulation. Although this time difference seems quite short, it is significant to decrease the exposure time to radiation for both the patient and operator, especially if we take into consideration that one operator implants approximately twenty CRT devices annually, the exposure time to radiation decreases by at least two and a half hours per year. In coronary catheter cannulation, moving with images with minimal contrast agent reduces the time required for the cannulation and decreases the fluoroscopy time. The main advantage of EP catheters is that they can easily pass through the valve structures that make it difficult to enter the coronary sinus. For two patients in our study, success was not achieved with the telescopic method, but EP catheters was successful, which may be due to coronary sinus valve structure. In addition, if intracardiac recordings can be obtained using an EP catheter, the cannulation time can be shortened even further. In our study, only the structural characteristics of the EP catheters were used without intracardiac recordings, which may have had an impact on time. On the other hand, the need for additional costs, personnel, and equipment in order to receive intracardiac recordings such as EP-tracer devices make it difficult for this method to be accessible and usable. Wang et al. [22] compared intra-cardiac cannulation methods through conventional methods and EP catheters and revealed that coronary sinus access was faster and the success was higher. Studying with and without intracardiac recordings with EP catheters may lead us to the best, easiest, and most practical method.

In patients with heart failure, kidney function is one of the important issues. The renal function of patients with congestive heart failure is affected due to many factors such as activation of the sympathetic nervous system, activation of the renin-angiotensin system, and an increase in anti-diuretic hormone (ADH) levels. Contrast agents increase the risk of contrast-induced nephropathy in patients with reduced renal function as well as increased heart failure [23]. In our study, the use of contrast media was not statistically significant, although the amount of contrast media was higher compared with the group in which coronary catheters were used. However, contrast nephropathy was not observed in our patient population. This is because uses less contrast than other invasive procedures and, therefore, did not cause chronic kidney disease in our cohort. In patients with normal renal function, coronary sinus cannulation using a coronary catheter can shorten the fluoroscopy time. However, the possibility of causing contrast nephropathy should not be underestimated, especially in patients with heart failure.

CRT procedures have a failure rate of 5–13%due to failure to perform coronary sinus cannulation and/or the absence of cardiac vena to place the lead. In our study, the implantation success rate was 97%. In the two failed cases, the coronary sinus was not cannulated due to anatomic difficulties. All methods for these patients were tried but because cannulation could not be performed, left system coronary angiography was performed and the coronary sinus ostium was visualized in the venous phase. Coronary sinus perforation is another important consideration in cannulation using coronary catheters and EP catheters; this risk should be kept in mind especially when catheters such as AL2 are used.

## Limitations

Our study had some limitations. The study population was relatively small and all CRTs were implanted by experienced electrophysiologists. Another limitation is the determination of random methods for patients without knowing the anatomy of the coronary sinus. The catheter selection in the methods is dependent on the operator's experience and preference, thus making it an important limitation of the study. Additionally, the radiation dose was not calculated. Lastly, the team conducting the study also reported the results. This is an important limitation given the possibilities for bias.

## Conclusion

This study is the first to compare electrophysiology catheter-mediated cannulation and coronary catheter-mediated cannulation for coronary sinus cannulation. We showed that coronary catheter-mediated cannulation reduced fluoroscopy time. This method can be used as a standard for cardiac resynchronization therapy.

## Supporting information

S1 Data The SPSS dataset of the study. (SAV)
